# PKR activation in mitochondrial unfolded protein response-mitochondrial dsRNA might do the trick

**DOI:** 10.3389/fcell.2023.1270341

**Published:** 2023-08-29

**Authors:** Eva Rath

**Affiliations:** Chair of Nutrition and Immunology, Technische Universität München, Freising-Weihenstephan, Germany

**Keywords:** mitochondrial unfolded protein response, inflammatory bowel diseases, doublestranded RNA, proteostasis, double-stranded RNA-activated protein kinase, integrated stress response, mitochondria, stress signaling

## 1 Introduction

Cells need to adapt constantly to internal and environmental changes ranging from normal physiological fluctuations to pathological alterations. Changes in the cellular demand can cause perturbations in different cellular compartments that in turn activate distinct signaling pathways to elicit transcriptional programs aiming at resolving the perturbation at the site of origin. In line, distinct adaptive responses to different types of “stresses” have been described for mitochondria. However, while responses to oxidative stress and hypoxia as well as events resulting in apoptosis are well understood, knowledge on signals, mediators, and targets employed in the response to disturbed mitochondrial proteostasis is still rudimentary (Ryan and Hoogenraad, 2007; Vogtle, 2021). Yet, protein aggregation has a significant impact on mitochondrial function and consequently, imbalances in mitochondrial proteostasis are implicated in ageing and are associated with a plethora of human diseases (Rath et al., 2018; Suomalainen and Battersby, 2018). The mitochondrial unfolded protein response (mtUPR or UPR^mt^) evoked by insufficient protein-folding capacity, accumulation of misfolded proteins or nondegradable protein aggregates in mitochondria, is a protective response to restore proteostasis. Upregulating nuclear-encoded mitochondrial chaperones and proteases as well as controlling mitochondrial RNA translation, mtUPR improves the mitochondrial folding environment, thus maintaining mitochondrial integrity (Munch, 2018). Although a growing number of players in mtUPR has been identified in the recent years, many open questions remain, including the identity of the initial signal, as well as unidentified molecular components to sense and mediate the retrograde signal to the nucleus (Vogtle, 2021). In 2011, we identified the double-stranded RNA (dsRNA)-activated protein kinase (PKR) as a signaling component of the mammalian mtUPR and demonstrated its disease-relevance for inflammatory bowel diseases (Rath et al., 2011), findings that have been confirmed by us and others (Jackson et al., 2020; Khaloian et al., 2020). However, we were not able to identify the signal that leads to PKR activation upon induction of mtUPR by expression of a mutant protein, ornithine transcarbamylase (OTC)Δ, that accumulates in a misfolded state in the mitochondrial matrix (Ryan and Hoogenraad, 2007; Rath et al., 2011). New findings by Kim et al. now indicate that PKR can be activated by mitochondrial RNA that exist as intermolecular dsRNA, in particular under stress conditions (Kim et al., 2018). These results contribute to a more comprehensive understanding of mitochondrial stress signaling and make it tempting to speculate that mtUPR-associated PKR activation is mediated via mitochondrial dsRNA.

## 2 Signaling disturbed mitochondrial proteostasis

Mitochondrial proteostasis can be used as a sensitive measure for cellular functionality, as it faces the unique challenge of coordinating import and processing of mitochondrial precursor proteins from the cytosol with the mitochondrial transcriptional and translational machinery to ensure the stoichiometric assembly of respiratory chain complexes (Ryan and Hoogenraad, 2007). Physiological triggers like fluctuating cellular energy demands, oxidative stress, and infections, can impair protein folding (Ron and Walter, 2007; Ryan and Hoogenraad, 2007), highlighted by the fact that mtUPR contributes to the dynamically regulated mitochondrial biogenesis program (Hood et al., 2006).

**FIGURE 1:**
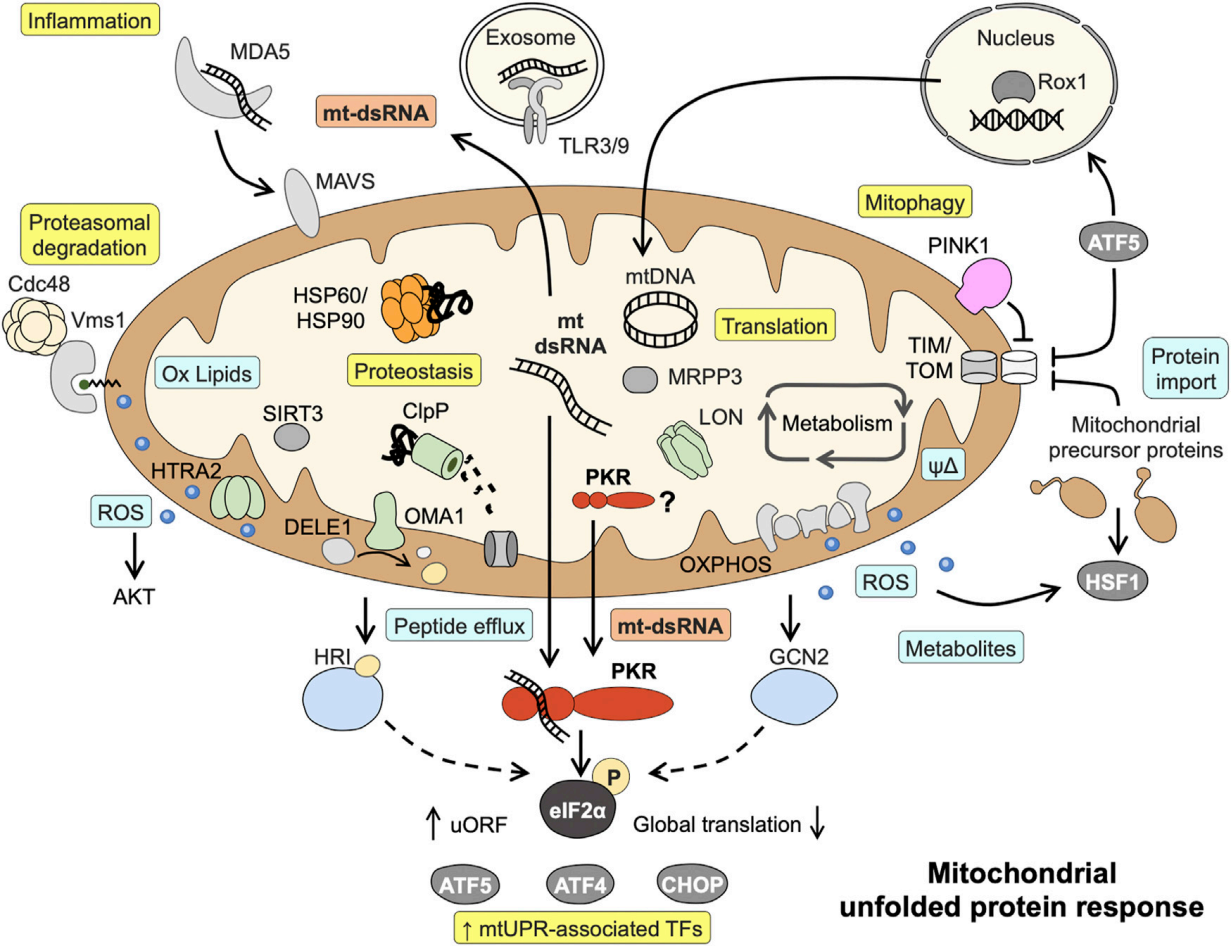
Known mitochondrial responses to disturbed proteostasis and proposed role of mitochondrial dsRNA and PKR in mtUPR signaling. Mitochondrial dsRNA might play a central role in activating the eIF2α kinase PKR and acts as danger signal capable of inducing inflammatory pathways. Light blue boxes = initial signals mediating mtUPR; Yellow boxes = endpoints of signaling. Details and abbreviation are given in the main text.

The quest to identify the initial sensor or signal activated by disrupted proteostasis has been complicated by the use of different model systems (yeast, *Caenorhabditis elegans*, different mammalian cell lines) and a large number of “stressors” used to evoke mtUPR signaling (Vogtle, 2021). Responses seem to be highly specific for organisms and stress triggers, and comparing the same stress trigger in metabolically different cells furthermore demonstrates that the initial metabolic state of the cell modulates mtUPR signaling (Mick et al., 2020). Depending on the stressor used (mainly chemical inhibitors of oxidative phosphorylation, and inhibitors or knockdown of proteases and chaperones), the following signals are mainly implicated in the initiation of mtUPR with different contributions: ROS and metabolites synthesized within mitochondria, peptide fragments derived from protease-mediated protein degradation and altered protein transport across the mitochondrial membranes (Figure 1) (Munch, 2018; Song et al., 2021). For example, ROS generated in yeast cells treated with respiratory chain inhibitors induce the generation of oxidized lipids (ergosterol peroxide). These serve as interaction partners of Vms1 in the outer mitochondrial membrane and recruit cofactors for the proteasome-mediated cytoplasmatic degradation of ubiquitylated outer membrane proteins (Nielson et al., 2017). In contrast, a proposed sensor for unspecific mitochondria-released peptides due to stress-induced proteolysis or the overall rate of efflux is still missing (Yano, 2017). However, the targeted cleavage of DELE1, a protein associated with the mitochondrial inner membrane, by proteases has been shown to give rise to a protein fragments activating the cytoplasmic kinase HRI (Fessler et al., 2020; Guo et al., 2020). Supporting the notion on differences and also convergence of mtUPR signaling, two different mitochondrial proteases were implicated in DELE1 cleave after CCCP (proton ionophore) or oligomycin (ATP synthase inhibitor) treatment, HTRA2 and OMA1, respectively (Fessler et al., 2020; Guo et al., 2020; Bi et al., 2023). The probably most famous signal for mitochondrial stress is blocked protein import via the TIM/TOM complexes, although *vice versa* the translocation of a nuclear protein (Rox1) to the mitochondrial matrix to protect the mtDNA and sustain translation upon mitochondrial perturbation has also been described (Poveda-Huertes et al., 2020). One current paradigm is that the mtUPR-associated transcription factor ATF5 (analogue to *C. elegans* ATFS-1) is regulated by dual localization (Nargund et al., 2012; Fiorese et al., 2016). During homeostasis, ATF5 is imported and degraded within mitochondria, but upon stress-induced impaired protein import into mitochondria, it translocates to the nucleus to induce the transcriptional mtUPR program. Consistent with a model of sensing mitochondrial protein import in a general way, pharmacological inhibition of the mitochondrial chaperone HSP90 leads to accumulation of mitochondrial protein precursors in the cytosol and a parallel release of mtROS into the cytosol, activating a cytosolic signaling cascade involving HSF1 (Sutandy et al., 2023). Similarly, it has been proposed that accumulation of newly synthesized PINK1 into the outer mitochondrial membrane as a consequence of collapsed mitochondrial protein import serves as a trigger for mitophagy (Jin and Youle, 2013; Fiesel et al., 2017) (Figure 1). Of note, the defects in mitochondrial protein import and quality control in these studies were not associated with mitochondrial depolarization. A proposed outcome of mtUPR signaling induced by impaired protein import into mitochondria is increased expression of mitochondrial chaperones and proteases that need to be imported into the mitochondrial matrix to fulfil their functions (Vogtle, 2021). This apparent contradiction suggests either that parallel, faster signals (partly) restore the mitochondrial protein import capacity before new nuclear-encoded mitochondrial precursor proteins are translated or a prioritized import of certain proteins into the mitochondria.

## 3 The mitochondrial unfolded protein response

Several axes of the mtUPR have been characterized that in part share components with other stress signaling pathways such as the integrated stress response (ISR), the endoplasmic reticulum unfolded protein response (erUPR), and the cytosolic heat shock response (HSR) (Rath et al., 2011; Munch, 2018; Sutandy et al., 2023). Most likely, the different mtUPR signaling axes act synergistically and with some redundancy. The current paradigm also comprises a cascade of events, beginning with local responses including translational regulation escalating to cell-wide responses comprising nuclear gene regulation if disturbances are more severe or affect a larger proportion of the cell´s mitochondria. Consequently, starting off as a protective response aiming to alleviate the protein-folding burden, mtUPR can result mitophagy in the case of severe or irreversible dysfunction (Munch, 2018; Samluk et al., 2019). With the exception of the intermembrane space (IMS) UPR axis, all axes are activated upon mitochondrial protein misfolding/aggregation in the mitochondrial matrix [reviewed in (Munch, 2018; Rath et al., 2018)]. (I) IMS mtUPR employs ROS-activated AKT to phosphorylate estrogen receptor- α (ERα) leading to transcriptional induction of NRF1 and the IMS-localized protease HTRA2. (II) The sirtuin axis acts via SIRT3, a protein deacetylase targeting FOXO3A causing its translocation to the nucleus to enhance transcription of ROS detoxification and mitophagy-associated genes. (III) The axis first described, the canonical mtUPR, results in upregulation of mitochondrial chaperones and proteases involving the transcription factors CHOP, CEBPβ, AP1, ATF4, and ATF5. (IV) A local translational mtUPR diminishes mitochondrial translation by LON-mediated degradation of the mitochondrial pre-RNA processing nuclease MRPP3 (Figure 1).

## 4 Eif2α phosphorylation and kinases in mtUPR

Various mitochondrial defects result in phosphorylation of eIF2α, a hallmark of the ISR, and also the canonical axis of mtUPR entails eIF2α phosphorylation (Rath et al., 2011; Quiros et al., 2017; Zurita Rendon and Shoubridge, 2018; Fessler et al., 2020; Mick et al., 2020). Phosphorylation of eIF2α results in global attenuation of cytosolic protein translation and selective translation of mRNAs containing upstream open reading frames (uORFs) such as CHOP, ATF4, and ATF5, thus underlining the central role of this event in mtUPR signaling. Four different kinases are known to phosphorylate eIF2α, the ER membrane-associated kinase PERK and the cytosolic kinases GCN2, HRI and PKR. With the exception of PERK, all kinases have been shown to be directly involved in mtUPR signaling in different experimental model systems. GCN2 responds to amino acid or glucose deprivation by binding to uncharged tRNAs as well as ROS, and ROS are required for GCN2 activation under mitochondrial stress (Baker et al., 2012). HRI is classically activated by heme deficiency, but an alternative way of activation by mtUPR signaling has been described (see above) (Fessler et al., 2020; Guo et al., 2020). Last but not least, PKR has first been characterized as kinase initiating immune response during infection by binding viral double-stranded RNAs (Gal-Ben-Ari et al., 2018). However, PKR has broad functions in sensing challenging cellular conditions and can be alternatively activated by its cellular protein activator of PKR (PACT) or endogenous dsRNAs such as small nucleolar RNAs during cell cycle or metabolic stress (Youssef et al., 2015; Chukwurah et al., 2021). Downstream, PKR posses several substrates including p53 and can modulate inflammatory and metabolic pathways including TNF signaling, JNK and NFκB activation, as well as insulin sensitivity (Gal-Ben-Ari et al., 2018). Consequently, PKR expression is induced by chemical inhibition of OXPHOS (Lee et al, 2020) and PKR activation is not only associated with IBD but also a hallmark of osteoarthritis and neurodegenerative diseases such as Alzheimer’s disease, Parkinson’s disease and Huntington’s disease (Bando et al., 2005; Rath et al., 2011; Ohno, 2014).

## 5 The riddle on PKR activation

Characterizing mtUPR signaling upon transfection of murine cells with OTCΔ in 2011, we unexpectedly discovered eIF2α phosphorylation as part of the signaling cascade. Importantly, we identified PKR to be responsible for phosphorylation of eIF2α. In this system, PKR is not only activated by disturbed mitochondrial proteostasis, but also a transcriptional target of the signaling. In line with previous publications showing the involvement of the MEK/JNK2 pathway and subsequent activation of AP1 in the model of OTCΔ-mediated mitochondrial stress induction (Horibe and Hoogenraad, 2007), we detected AP1 recruitment to the PKR promotor. Furthermore, we confirmed a role for the mitochondrial protease ClpP in the signaling pathway (Rath et al., 2011). Yet, searching for potential signals leading to PKR phosphorylation and thus activation we were only able to exclude several proposed mechanisms. Neither calcium signaling nor PACT were required for PKR activation/eIF2α phosphorylation. Additionally, using two different PKR knockout MEF cell lines with deletions in either the catalytic domain (C-PKR^−/−^) or the dsRNA-binding domain (N-PKR^−/−^), we found both domains to be required for mtUPR-induced phosphorylation of eIF2α (Rath et al., 2011; Rath, 2012).

## 6 Mitochondrial dsRNA and PKR activation

In contrast to mtDNA, a well-known danger-associated molecular pattern (DAMP) activating TLR and cGAS-STING pathways, mtRNA has only recently gained attention as cellular danger signal (Dhir et al., 2018; Lee J. H. et al, 2020; Grochowska et al., 2022). The circular mitochondrial genome is bidirectional transcribed as long polycistronic precursor transcript from both strands prior to processing into individual RNAs (Ojala et al., 1981). Thus, mtRNA from both strands of mtDNA can bind each other to form intermolecular dsRNA, that in turn can act as mtDAMP if released into the cytosol or the extracellular space (Kim et al., 2022). Consequently, mitochondrial dsRNAs (mt-dsRNAs) are implicated in triggering innate immune responses via MDA5 and TLR3 (Dhir et al., 2018; Lee J. H. et al, 2020; Kim et al., 2022) and also in disease-associated PKR activation (Kim et al., 2022; Yoon et al., 2022; Zhu et al., 2023). Actually, applying formaldehyde crosslinking, Kim et al. revealed that the majority of endogenous RNAs interacting with PKR is mt-dsRNA. Additionally, Kim et al. demonstrated that the abundance of mt-dsRNA and PKR activation is tightly regulated during cell cycle progression and under severe stress conditions (Kim et al., 2018; Kim et al., 2022).

The abundance of mt-(ds)RNA is determined by mtRNA synthesis and degradation and correlates with efflux into cytoplasm and PKR activation (Kim et al., 2018; Kim et al., 2022; Yoon et al., 2022; Zhu et al., 2023). With regard to disturbed mitochondrial proteostasis, two mechanisms linking mtUPR and mt-dsRNA generation/leakage into the cytoplasm seem likely, regulation of protease activity and/or ROS generation. Using an inhibitor of the mitochondrial chaperone HSP90 resulting in mitochondrial protein aggregate formation, it was shown that rapid degradation of the mitochondrial pre-RNA processing nuclease MRPP3 by the protease LON leads to defective pre-RNA processing and a stall in translation (Munch and Harper, 2016). These reversible processes could transiently increase the abundance of mtRNA and are in line with our results showing a role for mitochondrial proteases in mtUPR-induced PKR activation. Of note, the mtRNA encoding the ND5 locus is a preferred binding partner of PKR, and ND5 was shown to be a target of mitochondrial translational inhibition in the course of mtUPR (Munch and Harper, 2016; Kim et al., 2018). Furthermore, the transcription factor AP1/cJun, that we found to be activated by OTCΔ expression (Rath et al., 2011) has been shown to decrease mtDNA transcription by direct binding to mtDNA (Chae et al., 2013), indicating a potential feedback mechanism. On the other hand, mt-(ds)RNA decay might be affected by disturbances of mitochondrial proteostasis. MtRNA degradation takes place in the mitochondrial matrix and the IMS and involves, among others, the helicase SUV3 and the ribonuclease PNPase (encoded by *PNPT1*) (Luna-Sanchez et al., 2021). Loss of each of the proteins results in mt-dsRNA accumulation (Dhir et al., 2018; Pajak et al., 2019), but only PNPase seems to be involved in preventing mt-dsRNA efflux from mitochondria and downstream signaling including PKR activation (Dhir et al., 2018; Zhu et al., 2023). Next to PNPase, the release of mtRNA into the cytosol involves BAX/BAK pores, particularly upon mtDNA damage (Dhir et al., 2018; Tigano et al., 2021). Similarly, under severe stress conditions causing apoptosis, mitochondrial outer membrane permeabilization (MOMP) or disruption of mitochondrial membranes may lead to release of mtRNA to the cytosol and subsequent PKR activation (Kim et al., 2022). Vice versa, the mitochondrial chaperone HSP60, a target gene of mtUPR, is implicated in the retention of mt-dsRNAs in mitochondria, thereby reducing inflammatory signaling (Huang et al., 2022). Yet, PKR activation might also take place inside mitochondria, as a fraction of PKR is present in the mitochondrial matrix (Kim et al., 2018). In line, a proteomic study showed that PKR interacts with mitochondrial proteins, including HSP60 (Nakamura et al., 2015).

Overall, these findings suggest a model in which mt-dsRNA serves as signal sensed by PKR to integrate mitochondrial stress signaling into global cellular responses (Figure 1).

## 7 Conclusion and future directions

Some important questions remain, for example, how exactly disturbances in mitochondrial proteostasis might account for increased abundance and release of mt-dsRNA, if mt-dsRNA efflux involves active transport processes, or where exactly PKR activation takes place in the cell. It is likely that several signals are required for mt-dsRNA signalling, resembling other axes of mtUPR activation that have been shown to be dependent on multiple factors (Sutandy et al., 2023).

However, the findings by Kim et al. already shed new light on mitochondrial signaling and are a step towards a mechanistic and more holistic understanding of cellular responses toward mitochondrial disturbances (Monzel et al., 2023). It will be exciting to validate if mtUPR involves mt-dsRNA-initiated signaling and PKR activation. These data could be a framework to explore new targets for intervention in pathology-associated mitochondrial dysfunction.

## Figures and Tables

**Table:** 

**AKT**	Serine/threonine kinase
**AP1**	Activator protein 1
**ATF**	Activating transcription factor
**ATFS-1**	Stress activated transcription factor
**CCCP**	Carbonyl cyanide m-chlorophenyl hydrazone
**Cdc48**	Cell division cycle
**CHOP**	C/EBP homologous protein
**CEBPβ**	CCAAT/enhancer-binding protein beta
**ClpP**	Caseinolytic mitochondrial matrix peptidase
**DELE1**	DAP3-binding cell death enhancer 1
**eIF2α**	Eukaryotic translation initiation factor 2A
**FOXO3A**	Forkhead box O3
**GCN2**	General control nonderepressible 2
**HRI**	Heme-regulated inhibitor kinase
**HSF1**	Heat shock factor 1
**HTRA2**	High temperature requirement protein A2/Serine peptidase 2
**JNK2**	C-Jun Nh2-terminal kinase 2
**LON**	Serine protease
**MAVS**	Mitochondrial antiviral-signaling protein
**MDA5**	Melanoma differentiation-associated protein 5
**MRPP3**	Mitochondrial ribonuclease P protein 3
**ND5**	NADH dehydrogenase subunit 5
**NRF1**	Nuclear respiratory factor 1
**OMA1**	Overlapping with the m-AAA protease 1 homolog/Mitochondrial metalloendopeptidase
**OXPHOS**	oxidative phosphorylation
**PERK**	Protein kinase R-like endoplasmic reticulum kinase
**PINK1**	PTEN-induced kinase 1
**PNPase/*PNPT1***	Polyribonucleotide Nucleotidyltransferase 1
**ROS**	Reactive oxygen species
**Rox1**	Regulation by oxygen/Heme-dependent repressor of hypoxic genes
**SIRT3**	NAD-dependent protein deacetylase sirtuin-3
**SUV3**	Suv3 like RNA helicase
**TIM**	Translocase of the inner membrane
**TOM**	Translocase of the outer membrane
**TLR3**	Toll-like receptor 3
**Vms1**	VCP/Cdc48-associated Mitochondrial Stress-responsive
